# Supplementary material for: Effects of taping techniques on arch deformation in adults with pes planus: A meta-analysis
Source: PLoS One. 2021 Jul 2;16(7):e0253567. doi: 10.1371/journal.pone.0253567 (PMC8253385; doi:10.1371/journal.pone.0253567)
Supplement: S1 Table — (DOCX) [file pone.0253567.s002.docx]

| Abbreviation | Full name |
| --- | --- |
| NH | Navicular height |
| NDD | Navicular drop distance |
| ALD | Augmented low-Dye taping technique |
| MLD | Modified low-Dye taping technique |
| Fan-arch support | Fan-arch support medial longitudinal arch taping technique |
| FPI | Foot posture index |
| RCSP | Resting calcaneal stance position |

**S1 Table.** **Abbreviation key list.**
